# Comparison of Different Chemical and Mechanical Modalities for Implant Surface Decontamination: Activity against Biofilm and Influence on Cellular Regrowth—An *In Vitro* Study

**DOI:** 10.3389/fsurg.2022.886559

**Published:** 2022-09-30

**Authors:** Filippo Citterio, Elisa Zanotto, Gaia Pellegrini, Laura Annaratore, Anna Maria Barbui, Claudia Dellavia, Giacomo Baima, Federica Romano, Mario Aimetti

**Affiliations:** ^1^ Department of Surgical Sciences, Section of Periodontology, C.I.R. Dental School, Università di Torino, Turin, Italy; ^2^ Microbiology and Virology Unit, University Hospital City of Health and Science of Turin, Turin, Italy; ^3^ Department of Biomedical Surgical and Dental Sciences, Università degli Studi di Milano, Milan, Italy; ^4^ Department of Medical Sciences, Pathology Unit, Università degli Studi di Torino, Turin, Italy; ^5^ Pathology Unit, Candiolo Cancer Institute, FPO IRCCS, Candiolo, Italy

**Keywords:** peri-implantitis, dental implant, decontamination, biofilm, cellular growth, re-osseointegration

## Abstract

**Objectives:**

The aim of this *in vitro* study was to compare the efficacy of chemical and mechanical methods for decontamination of titanium dental implant surfaces previously infected with polymicrobial biofilms in a model simulating a peri-implant defect. Furthermore, the effect of each decontamination protocol on MG-63 osteoblast-like cells morphology and adhesion to the treated implants was assessed.

**Background:**

Peri-implantitis is a growing issue in dentistry, and evidence about implant surface decontamination procedures is lacking and inconclusive.

**Methods:**

A total of 40 previously biofilm-contaminated implants were placed into a custom-made model simulating a peri-implant defect and randomly assigned to five treatment groups: (C) control (no treatment); (AW) air abrasion without any powder; (ESC) air abrasion with powder of erythritol, amorphous silica, and 0.3% chlorhexidine; (HBX) decontamination with a sulfonic/sulfuric acid solution in gel; and (HBX + ESC) a combination of HBX and ESC. Microbiological analysis was performed on five implants per treatment group, and the residual viable bacterial load measured in log 10 CFU/mL was counted for each bacterial strain and for the total number of colonies. The remaining three implants per group and three noncontaminated (NC) implants were used to assess surface biocompatibility using a scanning electron microscope and a backscattered electron microscope after seeding with MG-63 cells.

**Results:**

A significant decontaminant effect was achieved using HBX or HBX + ESC, while no differences were observed among other groups. The percentage of implant surface covered by adherent MG-63 cells was influenced by the treatment method. Progressive increases in covered surfaces were observed in groups C, AW, ESC, HBX, HBX + ESC, and NC.

**Conclusions:**

A combination of mechanical and chemical decontamination may provide more predictable results than mechanical cleaning alone.

## Introduction

A bacterial biofilm is regarded as the etiologic agent of peri-implantitis in susceptible hosts (1). Therefore, aside from the correction of important risk factors, it is undoubted that the treatment of this disease is targeted at the effective removal of the dysbiotic biofilm (2). Nonsurgical treatment is usually the first step of implant surface decontamination, and it may be implemented with local or systemic antimicrobials. However, the morphology of the defect, the overall neighboring anatomy, the quality and shape of the prosthetic rehabilitation, and the macro- and microsurface characteristics of the fixture may offer protection to bacterial cells harboring the implant surface and may impair the effectiveness of nonsurgical treatment. Thus, in some cases, a surgical approach, providing better access to the implant surface, may facilitate the decontamination of the implant (3). A variety of chemical and/or mechanical methods have been tested for implant surface treatment, but none was found to be superior to others (4), and complete resolution of the disease is unlikely (5). Air powder abrasion has shown some advantages in terms of biofilm removal in some *in vitro* experiments (6–9); however, complete surface cleaning has not been achieved irrespective of the nonsurgical (10) or surgical (11) approach.

Another key aspect of implant surface decontamination is the impact of treatment modalities on the surface topography and chemical composition that may impair the re-osseointegration. In this regard, glycine powder seems to affect the biocompatibility of the fixture after treatment significantly (12), while sodium bicarbonate and a powder composed of erythritol, amorphous silica, and 0.3% chlorhexidine (ESC) have been proven not to interfere with osteoblast regrowth (12–14).

In recent years, new approaches have been proposed to treat biofilm-induced diseases also due to the concern regarding antibiotic resistance and the limited effectiveness of antimicrobials against biofilms. A novel topical sulfonic/sulfuric acid (HBX) solution has been developed. The sulfate components strongly absorb water from vital organic biofilm components, which results in instantaneous irreversible inactivation and denaturation of their biological function (15). The use of HBX alone has shown promising results for the treatment of acute periodontal abscesses and peri-implantitis (16–18).

Therefore, the aim of this study was to assess the decontamination potential of HBX followed by air abrasion with ESC on previously biofilm-contaminated implants in terms of residual viable bacterial load measured in log 10 CFU/mL in comparison with the treatment with HBX alone, ESC alone, and air abrasion without any powder and no treatment. Furthermore, the effect of each decontamination protocol on MG-63 osteoblast-like cell morphology and adhesion to the treated implant surfaces was assessed.

## Materials and Methods

**Figure 1:**
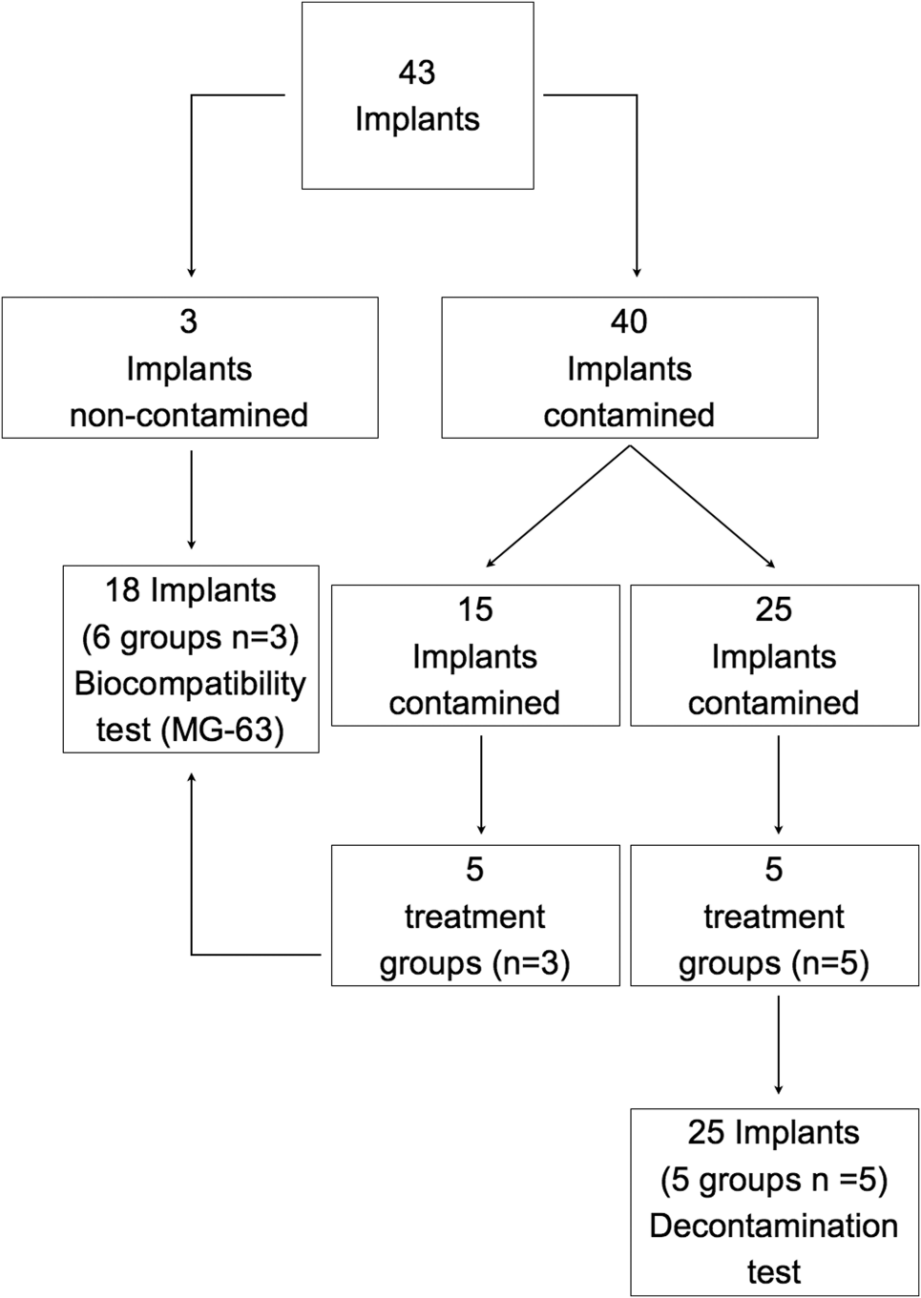
Schematic representation of the distribution of the implants into the different groups and study sections. C, control group—no treatment; AW, air abrasion without powder, ESC, air abrasion with powder of erythritol, amorphous silica, and 0.3% chlorhexidine; HBX, sulfonic/sulfuric acid solution in gel alone; HBX + ESC, sulfonic/sulfuric acid solution in gel followed by air abrasion with powder of erythritol, amorphous silica, and 0.3% chlorhexidine.

The protocol was approved by the Bioethical Committee of the University of Turin (Prot. No. 108391 21/02/2020). Forty-three sterile dental implants (OSSEOTITE XP Certain IOS IMPLANT 4.00** **mm × 1.50** **mm; BIOMET 3i LLC, Palm Beach Garden, FL, USA) were included in the study and allocated to different experimental procedures, as depicted in **Figure 1**.

### Implant Contamination

A vial polymicrobial biofilm was grown *in vitro* on 40 implants using the following commercially available strains:

 *Staphylococcus aureus* (ATCC25923),
 *Staphylococcus epidermidis* (ATCC49461),
 *Streptococcus anginosus* (ATCC33397),
 *Streptococcus salivarius* (ATCC13419),
 *Streptococcus mitis* (ATCC9811),
 *Fusobacterium nucleatum* (ATCC10953), and
 *Capnocytophaga ochracea* (ATCC27872).

Whole unstimulated saliva was collected from 10 periodontally healthy volunteers recruited among the dentistry students at C.I.R. Dental School, Turin, Italy. One examiner (FC) assessed their periodontal status by means of full-mouth periodontal examination. Periodontal health was defined as no sites with probing pocket depth ≥4** **mm and bleeding on probing on ≤2 interproximal sites with clinical attachment loss ≥2** **mm. Saliva was pooled, aliquoted, and stored at −20°C.

Biofilms were grown on 40 dental implants in a medium consisting of 60% whole unstimulated saliva and 40% brain heart infusion (BHI). In brief, each bacterial strain was separately cultured on CDC ANAEROBE+5% SB plates for 48** **h at 37°C in CO_2_ (*S. aureus*, *S. epidermidis*, *S. anginosus*, *S. salivarius*, *S. mitis*, and *C. ochracea*) or anaerobic conditions (*F. nucleatum*). Then, a bacterial suspension of 4 McF (1,200 × 106 CFU/mL) in BHI was prepared. Saliva aliquots were defrosted, and the bacteria contained in them were identified by means of matrix-assisted laser desorption ionization-time of flight (MALDI-TOF) (19).

Dental implants were then incubated in 3** **mL of defrosted pooled saliva in anaerobic conditions at room temperature for 4** **h to promote the formation of the acquired pellicle (20). Next, saliva was substituted with 1.8** **mL of defrosted pooled saliva, 1.2** **mL of BHI, and 602** **mL of mixed bacterial suspension (86** **µL of suspension of 4 McF per strain) that was removed and renewed after 16** **h. At 40** **h, the implants were washed and the culture medium was renewed. Incubated dental implants were repeatedly washed with sterile saline after 16, 20, 24, 40, 44, 48, and 64** **h in order to remove nonadhering bacteria. The total time of anaerobic incubation was 64** **h at 37.0°C (21).

### Model of Peri-Implantitis Defects

A model that simulated a crater-like peri-implant defect was created by inserting a hemisphere of 1** **cm diameter into dental impression material. After the impression material hardened, implants were placed into the model of the peri-implant defect with their flat bottom in contact with the bottom of the defect held by a metallic structure and impression material. The final peri-implantitis defect was composed of 5** **mm deep intrabony crater-like and 5** **mm deep suprabony components.

### Implants Decontamination

Forty contaminated implants were randomly assigned to five different groups, including four decontaminating procedures and one control group, by the use of a computer-generated random sequence of numbers:

Group C: no treatment;
Group AW: using only a spray of air and water coming from the air abrasive device;
Group ESC: air powder abrasion with ESC alone (Air-Flow Master, E.M.S. Electro Medical Systems GmbH, Munich, Germany; Air-Flow Plus Sub + Supragingival, E.M.S. Electro Medical Systems GmbH, Munich, Germany);
Group HBX: HBX alone (EPIEN MEDICAL, Saint Paul, MN, USA, containing 30%–60% sulfonated phenolic acid and 25%–30% sulfuric acid by weight); and
Group HBX + ESC: a combination of air powder abrasion with ESC and HBX.

Decontamination procedures were performed at the Section of Periodontology, C.I.R. Dental School, Turin, Italy. In ESC and HBX + ESC groups, an air abrasive system was used on the dental unit and set at a static water pressure of 4.5 bar and a static air pressure of 6 bar for each specimen. The cleansing time was set at 120 s per implant, with circumferential movements going all around the implant surface. Efforts were made in order to maintain the spray as perpendicular to the implant’s long axis as possible.

In groups EBX and HBX + ESC, HBX was applied for 20 s to the implant surface, proceeding from the most apical part of the defect to the most coronal part with circular movements. When the treatment procedure was the combination of ESC and HBX, the latter was applied before ESC. At the end of the treatment procedures, all the implants, including those of group C, were gently rinsed for 60 s with a sterile saline solution.

### Microbiological Tests

#### Quantification and Identification of Viable Bacterial Cells

After decontamination treatment, five implants per group (totally 25 implants) were randomly selected and transported to S.C. Microbiologia e Virologia U., AOU Città della Salute e della Scienza di Torino, Turin, Italy. The implants were placed in 15** **mL Falcon tubes, immersed in a 0.1% dithiothreitol (DTT) solution, and vortexed for 15** **min in order to remove the residual biofilm. Then, the implants were removed, and the DTT solution was centrifuged for 5** **min at 2,500** **rpm. The supernatant was eliminated, and the resulting cell suspension was serially diluted. About 20** **µL of suspended bacteria was collected, and aliquots of 10** **µL were plated in duplicate on blood agar plates supplemented with 5% defibrinated horse blood. For each dilution, two plates were incubated anaerobically (Gas Pak, Becton, Cockeysville, MD, USA) under controlled conditions using affiliated indicator strips. The other two plates were incubated aerobically at 37°C for 48** **h. The resulting colonies were counted (as CFU/mL) for each bacterial strain and the total number of colonies. *F. nucleatum* and *Propionibacterium acnes*, which were present in the pooled saliva, were counted in anaerobic conditions. The limit of detection was <1.0 **× **10^2^ CFU/mL. The limit of quantification was set at <2.5 **× **10^3^ CFU/mL. Quality controls (22–23) were performed during every relevant experimental stage. All counts were transformed into log_10_ CFU/mL. Bacteria were identified using MALDI-TOF.

### Biocompatibility Test

The remaining three implants per treatment group and the three noncontaminated/nontreated implants (group NC) (totally 18 implants) were transported to the Department of Medical Sciences, Pathology Unit, Università di Torino, Turin, Italy, for the biocompatibility test.

#### Osteoblast-Like Cell Regrowth on Treated Implants

Immediately after treatment, osteoblast-like cells (osteosarcoma cells, MG-63; ATCC CRL-1427; LGC Standards, Wesel, Germany) were seeded on top of implants. Before seeding the cells, 1.3** **mL of media was placed in each microplate well containing the implants. Then, 150** **µL of cell suspension, adjusted to 1.5 ×** **105 cells/mL, was pipetted in a meandering pattern above prepared specimens. The cells were cultured in Dulbecco’s modified Eagle’s medium (DMEM) with 10% fetal bovine serum (FBS) without phenol red and any antibiotics (to allow concomitant biofilm regrowth) at 37°C in a humidified atmosphere with 5% CO_2_ for 5 days, without media change. Cells were cultivated in tissue culture flasks (Eppendorf Italia Srl, Milan, Italy), were split at approximately 80% of confluence by a trypsin (0.05%)/EDTA (0.02%) solution (Sigma-Aldrich, Milan, Italy), and stopped with DMEM containing 20% FBS to attain an adequate number of cells.

#### Influence of Decontamination on MG-63 Growth

In order to assess the effect of each decontamination protocol on the MG-63 morphology and adhesion to the implant surface, after the incubation period, samples were fixed with 2.5% glutaraldehyde in buffered saline solution, dehydrated using a graded series of alcohol, dried, and observed using a scanning electron microscope (SEM) for morphological assessments and a backscattered electron microscope (BES) for semi-quantitative analysis (JEOL Ltd., Akishima, Tokyo, Nikon JCM-6000P, at 15** **kV). All implants were photographed by a blinded operator (GP) at the Department of Biomedical Surgical and Dental Sciences, Università di Milano, Milan, Italy. For each implant, (a) one photo at low magnification (20×) was taken to describe cell distribution on the implant surface; (b) six photos at a total magnification of 55× were taken in the area where cells had been seeded to perform semi-quantitative analysis; and (c) high-magnification photos (440× to 1,500×) were taken on randomly selected samples to assess the cellular morphology. The percentage of implant surface covered by adherent cells was calculated by the same blinded operator (GP) on 55× magnified photos using an image analysis system (Adobe, Photoshop CS5).

### Statistical Analysis

In order to assess the decontaminant effect of the different treatment methods, the viable CFU/mL was determined per identified bacterial species and per total bacterial counts. Thereafter, data were converted into the log scale to obtain a normal distribution. Due to the lack of homoscedasticity, the nonparametric Kruskal–Wallis test was performed. Multiple pairwise comparisons with Dunn’s test were performed to investigate intergroup differences for each bacterial strain.

The influence of decontamination treatment on cellular adhesion was analyzed by descriptive statistics. The percentage of implant surface covered by MG-63 cells was computed for all samples (*n* = 3) of each group; then, the mean and standard deviation were calculated for each group. The results were statistically analyzed using SPSS 24.0 (Chicago, IL, USA). The *p*-value was set at 0.05.

## Results

### Microbiological Test—Quantification of Viable Bacterial Cells

**Figure 2:**
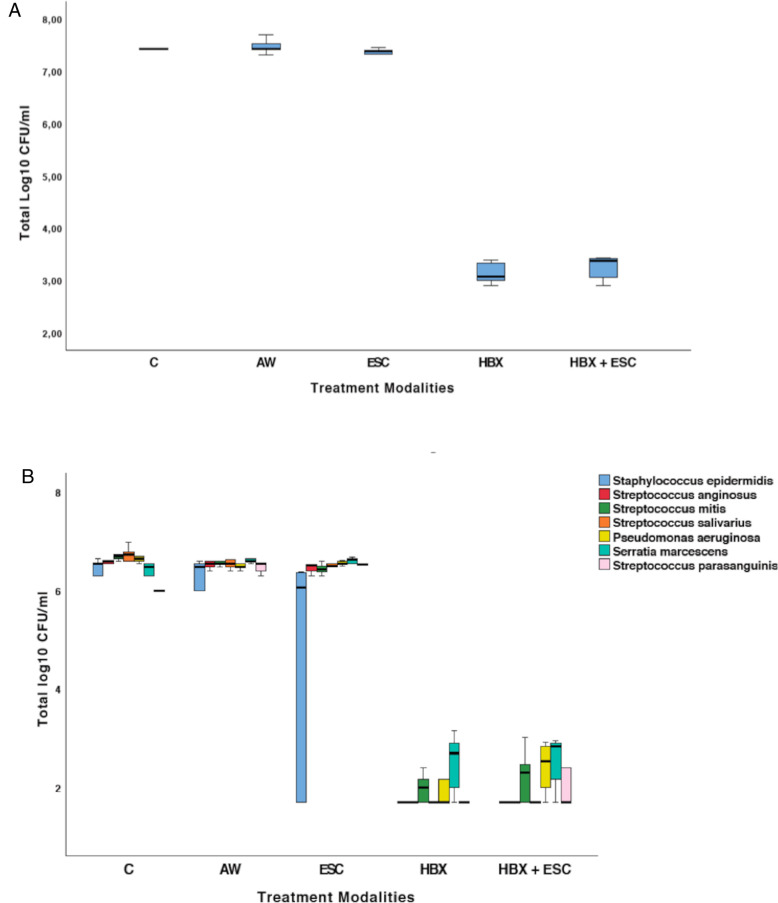
(**A**) Total viable log_10_ CFU/mL in the five treatment groups. (**B**) Changes in terms of log_10_ CFU/mL of *S. epidermidis*, *S. anginosus*, *S. mitis*, *S. salivarius*, *P. aeruginosa*, *S. marcescens*, and *S. parasanguinis* in the five treatment groups. C, control group—no treatment; AW, air abrasion without powder; ESC, air abrasion with powder of erythritol, amorphous silica, and 0.3% chlorhexidine; HBX, sulfonic/sulfuric acid solution in gel alone; HBX + ESC, sulfonic/sulfuric acid solution in gel followed by air abrasion with powder of erythritol, amorphous silica, and 0.3% chlorhexidine.

The effect of the five different decontamination methods on the viability of the implant-associated biofilm (log_10_ CFU/mL) is shown in **Figure 2A**. Means and standard deviations of log_10_ CFU/mL and logarithmic and percentage reductions per total bacterial counts are presented in **Table 1**. The Kruskal–Wallis test revealed that at least one group was different from the others (*p* = 0.001). Pairwise comparisons showed that the use of HBX [3.14 ± 0.21 log10(CFU/mL)] and the combination of HBX + ESC [3.24 ± 0.24 log_10_(CFU/mL)] was superior to group C [7.48 ± 0.12 log_10_(CFU/mL) *p* = 0.012 and *p* = 0.037, respectively] in reducing total bacterial counts. HBX also performed better than AW [7.48 ± 0.15 log_10_(CFU/mL) *p* = 0.018]. The differences between HBX + ESC and AW were on the threshold of statistical significance (*p* = 0.056). Group ESC [7.35 ± 0.10 log_10_(CFU/mL)] did not show statistically significant differences from any other group but showed a trend more similar to group C rather than to groups HBX and HBX + ESC.

**Table 1:** Mean, standard deviation, logarithmic, and percentage reduction compared to group C of total viable log_10_ CFU/mL in the five treatment modalities.

Treatment modality	Mean	SD	Log reduction	% reduction
C^a,c^	7.48	0.12	–	–
AW^b^	7.48	0.15	0	0
ESC	7.34	0.10	0.14	72.44
HBX^a,b^	3.14	0.21	4.34	99.99
HBX + ESC^c^	3.23	0.24	4.25	99.99

^a^ *p = 0.012.*

^b^ *p = 0.018.*

^c^ *p = 0.037.*

*C, control group—no treatment; AW, air abrasion without powder, ESC, air abrasion with powder of erythritol, amorphous silica, and 0.3% chlorhexidine; HBX, sulfonic/sulfuric acid solution in gel alone; HBX + ESC, sulfonic/sulfuric acid solution in gel followed by air abrasion with powder of erythritol, amorphous silica, and 0.3% chlorhexidine. Log reduction, logarithmic reduction of CFUs has been calculated compared to group C as the reference.*

MALDI-TOF identified the following bacteria in the pooled defrosted saliva: *Pseudomonas aeruginosa*, *Serratia marcescens*, *Streptococcus parasanguinis*, *Streptococcus faecalis*, *Klebsiella oxytoka*, *Granulicatella adiacens*, *P. acnes*, and *Micrococcus luteus*.

The bacteria detected on all implants of group C were *S. epidermidis*, *S. anginosus*, *S. mitis*, *S. salivarius*, *P. aeruginosa*, *S. marcescens*, and *S. parasanguinis*. Changes in terms of log_10_ CFU/mL for each strain are presented in **Figure 2B** and **Table 2**.

**Table 2:** Mean ± standard deviation viable log_10_ CFU/mL in the five treatment modalities for each bacterial species.

Bacterial species	C	AW	ESC	HBX	HBX + ESC
*S. aureus*	4.28 ± 2.37	3.54 ± 2.52	2.62 ± 2.06	<2.00	<2.00
*S. epidermidis*	6.47 ± 0.16^a^	5.46 ± 2.12	4.44 ± 2.50	<2.00^a^	<2.00^a^
*S.anginosus*	6.61 ± 1.00^b^	6.53 ± 0.08^c^	6.46 ± 0.11	<2.00^b,c^	<2.00^b,c^
*S. mitis/oralis*	6.72 ± 0.13^b,e^	6.63 ± 0.20^d^	6.44 ± 0.11	1.99 ± 0.30^b,d^	2.24 ± 0.56^e^
*S. salivarius*	6.74 ± 0.15^f^	6.60 ± 0.22	6.47 ± 0.19	<2.00^f^	<2.00^f^
*F. nucleatum*	2.59 ± 2.00	3.55 ± 2.54	2.64 ± 2.10	<2.00	<2.00
*C. ochracea*	2.62 ± 2.06	2.62 ± 2.06	<2.00	<2.00	<2.00
*P. aeruginosa*	6.71 ± 0.20^b,h^	6.56 ± 0.20	6.56 ± 0.05^g^	2.03 ± 0.53^b,g^	2.40 ± 0.54^h^
*S. marcescens*	6.62 ± 0.27	6.66 ± 0.14^j,k^	6.57 ± 0.12^i^	2.49 ± 0.62^i,j^	2.52 ± 0.56^k^
*S. parasanguinis*	6.08 ± 0.18	6.55 ± 0.25^l,m^	6.59 ± 0.14^f,n^	<2.00^f,l^	1.98 ± 0.38^m,n^
*S. faecalis*	2.56 ± 1.92	3.48 ± 2.44	<2.00	<2.00	<2.00
*K. oxytoka*	2.56 ± 1.92	3.48 ± 2.44	<2.00	<2.00	<2.00
*G. adiacens*	<2.00	3.45 ± 2.40	<2.00	<2.00	<2.00
*P. acnes*	<2.00	<2.00	<2.00	<2.00	<2.00
*M. luteus*	<2.00	2.56 ± 1.92	<2.00	<2.00	<2.00

^a^ *p = 0.019.*

^b^ *p = 0.003.*

^c^ *p = 0.047.*

^d^ *p = 0.039.*

^e^ *p = 0.008.*

^f^ *p = 0.004.*

^g^ *p = 0.044.*

^h^ *p = 0.017.*

^i^ *p = 0.045.*

^j^ *p = 0.019.*

^k^ *p = 0.022.*

^l^ *p = 0.006.*

^m^ *p = 0.029.*

^n^ *p = 0.020.*

*C, control group—no treatment; AW, air abrasion without powder; ESC, air abrasion device with powder of erythritol, amorphous silica, and 0.3% chlorhexidine; HBX, sulfonic/sulfuric acid solution in gel alone; HBX + ESC, sulfonic/sulfuric acid solution in gel followed by air abrasion with powder of erythritol, amorphous silica, and 0.3% chlorhexidine.*

### Biocompatibility Test—Influence of Decontamination on MG-63 Morphology and Adhesion

**Figure 3:**
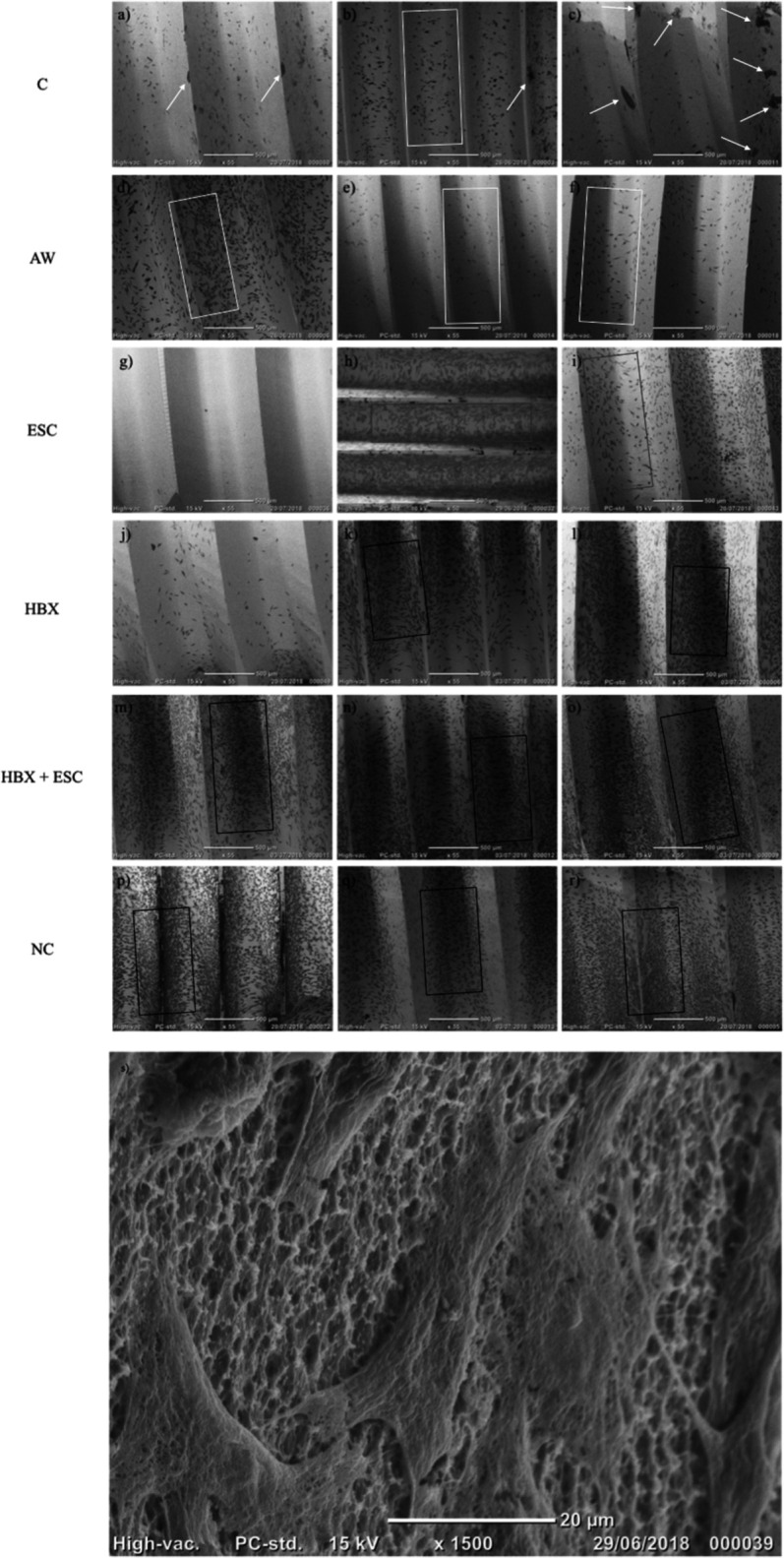
55× microphotographs of the implants from different experimental groups. White arrows, bacterial aggregates; white boxes, spread cells between implant threads; red boxes; non densely packed cells; black boxes, densely packed cells. C, control group – no treatment (**A–C**); AW, air-abrasive device without powder (**D–F**), ESC, air-abrasive device with powder of erythritol, amorphous silica and 0.3% chlorhexidine (**G–I**), HBX, sulfonic/sulfuric acid solution in gel alone (**J–L**), HBX + ESC, sulfonic/sulfuric acid solution in gel followed by air-abrasive device with powder of erythritol, amorphous silica and 0.3% chlorhexidine (**M–O**); NC, non contaminated, non treated implants (**P–R**). (**S**) Details of MG-63 cells adhering on a previously contaminated and subsequently treated implant surface (1,500×).

In morphological analysis by SEM, for all groups, cells appeared housed on the implant surface, with clear cytoplasmic extensions that allow the connection between cells as well as adhesion to the rough surface (**Figure 3S**). No functional orientation was observed in any group. In BES analysis, differences between groups were found in cellular distribution. In groups C and AW, pictures showed spread cells distributed mainly among implant threads (**Figure 3B,D–F**, white boxes). In group C, bacterial aggregates were visible (**Figure 3A–C**, white arrows). In the ESC group, cells covered the implant surface homogeneously but were not densely packed (**Figure 3H,I**, red boxes). In one specimen, no cell was visible (**Figure 3G**). In groups HBX, HBX + ESC, and NC, cells were more densely packed on the implant surface (**Figure 3K–R**, black boxes). However, in one specimen of group HBX, few cells covered the implant surface (**Figure 3J**). Semi-quantitative analysis revealed a trend toward an increasing percentage of implant surface covered by adherent cells from group C to groups HBX + ESC and NC (**Table 3**).

**Table 3:** Mean and standard deviation of the percentage of implant surface covered by MG-63 cells in the five different treatment groups and noncontaminated implants.

Treatment modality	Mean (%)	SD (%)
C	7.41	4.75
AW	12.41	4.38
ESC	24.11	6.72
HBX	33.55	11.33
HBX + ESC	51.69	9.55
NC	60.13	9.34

*SD, standard deviation; C, control group—no treatment; AW, air abrasion without powder; ESC, air abrasion with powder of erythritol, amorphous silica, and 0.3% chlorhexidine; HBX, sulfonic/sulfuric acid solution in gel alone; HBX + ESC, sulfonic/sulfuric acid solution in gel followed by air abrasion with powder of erythritol, amorphous silica, and 0.3% chlorhexidine; NC, noncontaminated, nontreated implants.*

## Discussion

In the present *in vitro* model, treatment with HBX either alone or in combination with ESC provided a significant decontaminant effect on previously contaminated implants, while no differences were observed between the groups receiving other treatments. Moreover, it was observed that the percentage of implant surface covered by adherent MG-63 cells after 5 days of incubation progressively increased through groups C, AW, ESC, HBX, HBX + ESC, and NC.

The treatment goal against peri-implantitis is to arrest the inflammatory process and possibly favor re-osseointegration, providing long-term stable results. If we assume that peri-implantitis is initiated and exacerbated by bacteria, then the biofilm removal becomes essential (24). Irrespective of the decontamination modality, nonsurgical treatment seldom results in the elimination of the disease, and there is a clear tendency for recurrence (2, 5, 24). Thus, when nonsurgical treatment is unable to provide satisfactory results, additional surgical treatment is suggested. For this reason, we decided to test different treatment modalities in an *in vitro* situation simulating the clinical conditions of surgical regenerative treatment, which instead has been proven somehow effective (2, 25, 26).

Moreover, the outcomes of regenerative therapy of peri-implantitis have been proven to be influenced by a variety of factors (27), such as the defect (28) and implant surface characteristics. For this reason, we opted to test the decontamination modalities in a circumferential defect with no dehiscence and on moderately rough surfaces since these conditions seem to be prerequisites for successful regenerative treatment.

In recent years, the most tested antimicrobial agents have been citric acid (CA), chlorhexidine (CHX), and hydrogen peroxide (H_2_O_2_). CA has demonstrated potential against single- and multispecies biofilms on titanium surfaces (29–31). However, it has never been tested against mature biofilms, and it often does not exceed the efficacy of saline rinses. CHX has shown bactericidal effect against early and mature biofilms, but no cleaning properties *per se* (30–32). H_2_O_2_ has a moderate to good bactericidal effect but no obvious cleaning properties (30–33). Interestingly, in the present research, HBX has been proven able to produce a significantly greater reduction of viable bacteria compared to group C which received saline rinses. This could be explained by the antibiofilm properties of HBX that denaturates the organic components of the biofilm and desiccates the biofilm matrix, weakening its bonding to the titanium surface (15).

Moreover, if we take into consideration that mechanical debridement with air abrasive devices has been proven to leave a consistent amount of untouched implant surface in conditions simulating surgical access (11), we can assume that disinfection of titanium surfaces by mechanical means only might not be adequate. This finding is in line with previous studies (34, 35), which concluded that mechanical debridement alone was insufficient due to the complex implant surface topographies and claimed for a combination of mechanical and chemical modalities of implant surface decontamination. In the current study, ESC did not differ in terms of residual viable log_10_ CFU/mL for groups receiving no treatment. This is in contrast with the conclusions of a systematic review that found the *in vitro* cleaning efficacy of air-powder abrasive devices to be consistent (36). In general, studies using sodium bicarbonate, glycine, or ESC *in vitro* reported more than 84% removal of bacteria or bacterial byproducts irrespective of the surface type (8, 9, 14). Conversely, in the present research, ESC failed to reduce the bacterial load significantly, probably because the model of the peri-implant defect and the screw-shaped implants partially impeded its direct action on the biofilm. The studies reporting promising results for air-powder abrasive devices were, in general, performed either on titanium disks (9, 13, 14, 37, 38) or on implants without a peri-implantitis defect model (8), where the air abrasive devices could easily reach the implant surface.

The assessment of residual biofilm on treated implants has been performed using DTT, which has been proven as effective as sonication for the detection of biofilm-associated bacteria (39).

For the biocompatibility test, MG-63 osteoblast-like cells were used as they have often been employed as an osteoblastic model to study cell viability, adhesion, and proliferation on titanium surfaces. Within the limitation of this study, it has been observed that different treatment modalities have heterogeneous impacts on MG-63 cell proliferation. Semi-quantitative analysis showed that HBX and HBX + ESC might reduce the bacterial load to an extent, which may render the previously contaminated implant surfaces as biocompatible as the noncontaminated controls. AW and ESC showed a lower percentage of the covered implant surface. This is consistent with the results obtained in the first part of the experiment, where it was demonstrated that neither AW nor ESC was able to significantly reduce the bacterial load on contaminated implants. Despite being in contrast with previous findings (40), the observed trend suggests that the decontamination activity of the treatments may be directly related to the cellular growth on the implant surface. However, a direct comparison between the present research and other studies on the biocompatibility of implants after treatment is still difficult due to methodological differences. Nonetheless, as we observed that HBX did not prevent MG-63 cells from colonizing the implant surface and did not alter cell morphology, our findings support the fact that HBX may have no or limited cytotoxic activity compared to other decontaminants such as CHX or CA (41, 42).

A limitation of the present study is that the obtained biofilm cannot be compared with biofilms at diseased peri-implant sites, presenting deepened pockets with the microbiota of different quantities and qualities. *S. aureus* and *S. epidermidis* have been selected as they produce consistent amounts of exopolysaccharides and favor biofilm formation; S*. anginosus*, *S. salivarius*, *S. mitis*, *F. nucleatum*, and *C. ochracea* are the most common bacteria isolated from oral microflora and have been recognized as the main primary and secondary colonizers of dental and implant surfaces (43–45). Importantly, *C. ochracea* contributes to early plaque formation by being an intermediate physical link between several *Streptococcus* species and *F. nucleatum* by the release of a diffusible molecule that plays a significant role in the formation of biofilm by both bacterial species in a synergistic manner (46). However, in the present study, *F. nucleatum* and *C. ochracea* were seldom detectable, probably due to the conditions of biofilm growth and maturation. Another limitation of the present study is the degree of maturation of the *in vitro* biofilm, as more mature biofilms may be more resistant to chemical decontaminants (47, 48). In fact, it has been shown that 3-week-old biofilms, characterized by a subgingival microbiota comprising mainly Gram-negative and anaerobic bacteria, are resistant to the chemical agents commonly used in dental practice (32).

In conclusion, within the specific conditions and limitations of this *in vitro* study, it has been demonstrated that a significant decontaminant effect on moderately rough implants was achieved using the sulfonic/sulfuric acid solution in a gel. No differences were shown between the groups receiving other treatments. Moreover, treatment with HBX and the combination of HBX and ESC was able to reduce the contamination of the implants to a level that did not interfere with MG-63 cell growth on the decontaminated implants. These findings prompt further investigations into dental implant decontamination using chemical agents. A combination of physical and chemical therapies may provide more predictable results in the future.

## Data Availability

The raw data supporting the conclusions of this article will be made available by the authors without undue reservation.
